# Effect of short- and medium-chain fatty acid mixture on polyhydroxyalkanoate production by *Pseudomonas* strains grown under different culture conditions

**DOI:** 10.3389/fbioe.2022.951583

**Published:** 2022-07-25

**Authors:** Karolina Szacherska, Krzysztof Moraczewski, Sylwester Czaplicki, Piotr Oleskowicz-Popiel, Justyna Mozejko-Ciesielska

**Affiliations:** ^1^ Department of Microbiology and Mycology, Faculty of Biology and Biotechnology, University of Warmia and Mazury in Olsztyn, Olsztyn, Poland; ^2^ Institute of Materials Engineering, Kazimierz Wielki University, Bydgoszcz, Poland; ^3^ Department of Plant Food Chemistry and Processing, Faculty of Food Sciences, University of Warmia and Mazury in Olsztyn, Olsztyn, Poland; ^4^ Water Supply and Bioeconomy Division, Faculty of Environmental Engineering and Energy, Poznan University of Technology, Poznan, Poland

**Keywords:** biopolymers, medium-chain fatty acids, polyhydroxyalkanoates, *Pseudomonas* sp., short-chain fatty acids

## Abstract

Short- and medium-chain fatty acids (SMCFAs) derived from the acidogenic anaerobic mixed culture fermentation of acid whey obtained from a crude cheese production line and their synthetic mixture that simulates a real SMCFA-rich stream were evaluated for polyhydroxyalkanoate (PHA) production. Three individual *Pseudomonas* sp. strains showed different capabilities of growing and producing PHAs in the presence of a synthetic mixture of SMCFAs. *Pseudomonas* sp. GL06 exhibited the highest SMCFA tolerance and produced PHAs with the highest productivity (2.7 mg/L h). Based on these observations, this strain was selected for further investigations on PHA production in a fed-batch bioreactor with a SMCFA-rich stream extracted from the effluent. The results showed that PHA productivity reached up to 4.5 mg/L h at 24 h of fermentation together with the ammonium exhaustion in the growth medium. Moreover, the PHA monomeric composition varied with the bacterial strain and the type of the growth medium used. Furthermore, a differential scanning calorimetric and thermogravimetric analysis showed that a short- and medium-chain-length PHA copolymer made of 3-hydroxybutyric, -hexanoic, -octanoic, -decanoic, and -dodecanoic has promising properties. The ability of *Pseudomonas* sp. to produce tailored PHA copolymers together with the range of possible applications opens new perspectives in the development of PHA bioproduction as a part of an integrated valorization process of SMCFAs derived from waste streams.

## 1 Introduction

The negative impact of petrochemical plastics on the environment has prompted a growing interest in environmentally friendly alternative materials. Polyhydroxyalkanoates (PHAs) are especially attractive because of their unique material properties such as biodegradability, non-toxicity, and biocompatibility and the similarity of their physical properties to those of synthetic polymers (Zubairi et al., 2016). Moreover, PHAs have many advantages over petrochemical-derived polymers, such as hydrophobicity, thermoplastic processability, relatively high melting point, and optical purity. They are natural polyesters synthesized by various microorganisms under unbalanced growth conditions as intracellular energy compounds in the form of granules in the cytoplasm (Akaraonye et al., 2010). Generally, PHAs are classified according to the carbon-chain-length of the constituent monomers into short-chain-length (PHA_SCL_) containing 3–5 carbon atoms in the molecule, medium-chain-length (PHA_MCL_) with 6–14 carbon atoms in the molecule, and long-chain-length (PHA_LCL_) which contains more than 15 carbon atoms (Tan et al., 2014). It is known that the monomeric composition of PHAs depend mainly on the carbon source used for bacterial culture and the bacterium that is able to synthesize and accumulate PHAs (Koller and Braunegg, 2015). The applicability of bacterial PHAs is directly related to their properties. PHA_SCL_, such as poly-3-hydroxybutyrate [P(3HB)], shows high crystallinity, stiffness, and brittleness with poor elastic properties, limiting its use as a stable material. PHA_MCL_ shows more favorable and useful properties. They are elastomers with a low degree of crystallinity, melting point, and tensile strength as well as high elongation to break. They are synthesized mainly by bacteria of the *Pseudomonas* genus. Due to their useful properties, PHAs have great potential in biomedical, agricultural, and industrial applications (Tan et al., 2021). Nevertheless, the commercialization of PHAs in the world market is currently limited, mainly due to the high production costs compared to synthetic polymers. Despite the fact that the fermentation technology and polymer extraction process have considerably improved over the past decade, the cost of the carbon source still accounts for half of the cost of producing PHAs. Consequently, there is a growing need to develop novel microbial processes using renewable and inexpensive carbon sources. Short and medium chain fatty acids (SMCFAs) generated by acidogenic anaerobic mixed culture fermentation (MCF) are an interesting alternative to the expensive substrates. Recycling waste into desirable and valuable products is a revolutionary path in waste management and an environmentally friendly process (Martinez et al., 2015). Various renewable wastes converted into SMCFAs such as sugarcane molasses (Albuquerque et al., 2007), paper mill wastewater (Bengtsson et al., 2008), olive oil wastewater (Beccari et al., 2009), food waste (Venkateswar Reddy and Venkata Mohan, 2012; Aremu et al., 2021; Thomas et al., 2022), and pea shells (Patel et al., 2012) have been used to produce environmentally friendly PHAs. In contrast, few scientific publications have evaluated the potential of pure bacterial cultures to convert SMCFA-rich streams from MCF using organic waste into PHAs (Venkateswar Reddy et al., 2012; Cerrone et al., 2014; Martinez et al., 2015; Vu et al., 2021). It should also be mentioned that none of these studies evaluated, in detail, the properties of the extracted PHAs.

An important aspect in the cost-effective production of PHAs is also the selection of a microorganism that has the ability to efficiently intracellularly accumulate this biopolymer using renewable carbon sources. Bacteria belonging to the *Pseudomonas* genus meet these criteria by transforming post-fermentative carbon sources into multipurpose biopolymers. They are also characterized by fast biomass growth, low production requirements, easy adaptation, and tolerance to oxidative stress (Mozejko-Ciesielska et al., 2019). *Pseudomonas* sp. Have been characterized as the producers, usually producing mcl-PHA rather than scl-PHA (Solaiman and Ashby, 2005). The synthesis of PHAs from SMCFAs has been described for several *Pseudomonas* spp., including: *Pseudomonas* sp. ST2 (Reddy et al., 2008; Munir and Jamil, 2018), *Pseudomonas* sp. H9 (Liu et al., 2021), *P. putida* KT2440 (Kourmentza et al., 2009; Cerrone et al., 2014; Yang et al., 2019), *P. otitidis* (Venkateswar Reddy et al., 2012), *P. pseudoflava* (Venkateswar Reddy et al., 2017), *P. palleronii* (Venkateswar Reddy et al., 2017), and *P. oleovorans* (Aremu et al., 2021). To the best of our knowledge, none of these studies described the properties of the extracted scl-mcl-PHA copolymers.

In recent years, the amount of organic and biomass waste has been steadily increasing. Currently, sewage sludge is a burden for municipal wastewater treatment plants (Lad et al., 2022). Moreover, acid whey, a by-product of Greek yoghurt, cottage cheese production, and the like, poses a risk to the ecosystem and its disposal is associated with a fee (Erickson, 2017). Therefore, SMCFAs derived from sludge and acid whey are attractive raw materials for biopolymer synthesis by microorganisms, and this makes the bioprocess more environmentally friendly. This is the first study that reports about SMCFAs from the anaerobic mixed culture fermentation of acid whey, which is available worldwide, to produce unique scl-mcl PHAs. Three strains belonging to *Pseudomonas* genera were first evaluated to study their capability of producing PHAs while they grew on different media supplemented with a synthetic mixture of SMCFAs, that simulates a real SMCFA-rich stream, as the only carbon sources. Then, the most effective *Pseudomonas* sp. strain was cultured with SMCFAs derived from whey wastewater fermentation effluent. The extracted PHAs were characterized using GC/MS, FTIR, DSC, and TG to assess the application potential of these materials.

## 2 Materials and methods

### 2.1 Microorganisms and inoculum preparation

*Pseudomonas* sp. Gl01 and *Pseudomonas* sp. Gl06 strains were isolated from mixed microbial communities and belong to the Culture Collection of the Department of Microbiology and Mycology, University of Warmia and Mazury in Olsztyn. They were previously described as strains capable of synthesizing and accumulating PHA_MCL_ (Ciesielski et al., 2010). The *Pseudomonas antarctica* (DSM 15318) used in the study was purchased from the German Collection of Microorganisms and Cell Cultures GmbH at the Leibniz Institute.

### 2.2 Culture condition and carbon sources

Seed cultures were grown in lysogeny broth (10 g/L tryptone, 5 g/L yeast extract, 10 g/L NaCl) at 30°C, shaking at 150 rpm for 16 h. Then, the precultures were transferred to three different non-limited and nitrogen-limited mineral salt media (MSM): (MSM 1) 12.8 g/L Na_2_HPO_4_·7H_2_O, 3 g/L KH_2_PO_4_, 10 g/L NH_4_Cl, 0.5 g/L NaCl (Poblete-Castro et al., 2012); (MSM 2) 12.8 g/L Na_2_HPO_4_, 3 g/L KH_2_PO_4_, 10 g/L (NH_4_)_2_SO_4_; (MSM 3) 3.5 g/L Na_2_HPO_4_, 5 g/L KH_2_PO_4_, 10 g/L (NH_4_)SO_4_. Nitrogen-limited MSM consisted of 1 g/L of nitrogen source in each media. The media were supplemented with 1 g/L of MgSO_4_·7H_2_O and 2.5 ml/L of trace element solution consisting of (per liter): 20 g FeCl_3_·6H_2_O, 10 g CaCl_2_·H_2_O, 0.03 g CuSO_4_·5H_2_O, 0.05 g MnCl_2_·4H_2_O, and 0.1 g ZnSO_4_·7H_2_O dissolved in 0.5N HCl. The pH of each culture was adjusted to 7.0 by adding 1N NaOH and 1N HCl. All MSM components were dissolved in water and then autoclaved at 121°C. An MgSO_4_·7H_2_O solution was sterilized and added separately. The MSM were supplemented with 20% (v/v) of a mixture of synthetic SMCFAs (SMCFA_synthetic_-rich stream) or 20% (v/v) of SMCFAs extracted from mixed culture fermentation of acid whey (SMCFA_extracted_-rich stream). SMCFA_synthetic_-rich stream is a mixture of synthetic carboxylic acids composed of 2.85 g/L of acetic acid, 9.86 g/L of butyric acid, 0.16 g/L of valeric acid, and 3.05 g/L of caproic acid. The effluent for PHA production was taken from the acidogenic anaerobic mixed culture fermentation of acid whey obtained from a crude cheese production line (Duber et al., 2018). It was taken from a steady-state phase and contained (except for organic acids mentioned previously) 9.31 g/L lactic acid and 1.64 g/L ethanol.

The *Pseudomonas s*pp. strains were cultured in 250-ml Erlenmeyer flasks in a rotary shaker at 150 rpm, at 30°C for 48 h. Fermentation shake flasks were inoculated with 5% v/v of the precultures. The carbon source was added at the beginning of the cultivations. Three replicate cultivations were carried out. The bacterial cells were harvested at 48 h to determine the cell dry mass, PHA concentration, and composition.

### 2.3 Bioreactor fermentation

The preliminary cultivations in shake flasks led to the selection of MSM, consisting of 12.8 g/L Na_2_HPO_4_, 3 g/L KH_2_PO_4_, 1 g/L (NH_4_)_2_SO_4_ that supported the best PHA productivity, and *Pseudomonas* sp. GL06 as the most effective PHA producer.

The PHA production of the selected *Pseudomonas* sp. strain was conducted at 30°C with an aeration rate of 4 L/min in a 7 L bioreactor (Biostat A, Sartorius, Germany) equipped with a pH controller. The pH-value was maintained at seven through the modulated addition of concentrated 1N NaOH and 1N HCl. The temperature was maintained by a thermostatic jacket. The dissolved oxygen was monitored during the whole cycle with an O_2_ electrode (InPro 6800, Mettler Toledo GmbH, Switzerland) and was maintained at 50% air saturation by adjusting the agitation rate from 300 to 1,000 rpm automatically. Total fermentation time was 48 h. The inoculation size was 10% (v/v). The mineral salt medium was supplemented with a total of 20% (v/v) SMCFAs-extracted. The substrate was added in two portions: first at the beginning of the cultivation and then after 8 h of fermentation. A concentrate solution (Sigma Aldrich) was used as an antifoam in response to the antifoam controller. The samples of the culture broth were removed at several time intervals for analysis of biomass concentration, ammonium and phosphate concentration, PHA yield, the composition, and concentration of PHA monomers.

### 2.4 Analytical procedures

To measure cell dry mass (CDM), 100 ml of culture broth was centrifuged at 11.200 *×* g for 10 min and then lyophilized for 24 h using a Lyovac GT2 System (SRK Systemtechnik GmbH). Ammonium and phosphorus concentration were determined spectrophotometrically using a Hach Lange DR 2800 spectrophotometer (Hach Lange, Düsseldorf, Germany) as well as the LCK338 and LCK350 kits according to the manufacturer’s instructions, respectively. Quantitative and qualitative analysis of the obtained biopolymers was performed. Biopolyesters were extracted by shaking lyophilized cells in chloroform at 50°C for 3 h. Then the mixture was filtered through No. 1 Whatman filter paper to remove the cellular debris. Biopolymers dissolved in chloroform were purified by precipitation with a 70% cold methanol solution and then allowed to evaporate at room temperature. The PHA content (% of CDM) was defined as the percentage of the ratio of biopolymer concentration to total cell concentration.

### 2.5 PHA analyses

#### 2.5.1 Gas chromatography coupled with mass spectrometry

To evaluate the PHA composition, the lyophilized cells were analyzed by gas chromatography coupled with mass spectrometry (GC-MS QP2010 PLUS, Shimadzu, Japan) according to the method described by Możejko-Ciesielska and Pokój (2018). As a first step, analyzed samples were suspended in a chloroform-methanol-sulfuric acid mixture (100/97/3, v/v/v), and the methylation process was performed by heating the vials at 100°C for 20 h. After that, the sulfuric acid was neutralized with Na_2_CO_3_ and the resulting mixture was dried with anhydrous Na_2_SO_4_. After filtration, the methyl esters were analyzed using a BPX70 (25 m *×* 0.22 mm *×* 0.25 mm) capillary column (SGE Analytical Science, Victoria, Australia) with helium as a carrier gas at a flow rate of 1.38 ml/min. The column was heated from 80 to 240°C at a rate of 10°C/min. The interface and ion source temperatures of GC-MS were set at 240°C, and the electron energy was set at 70 eV. The total ion current (TIC) mode was used in 45–500 m/z range. Pure standards of methyl 3-hydroxybutyrate, 3-hydroxyvalerate, 3-hydroxyhexanoate, -octanoate, -nonanoate, -decanoate, -undecanoate, -dodecanoate, -tetradecanoate, and -hexadecanoate were purchased from Larodan Fine Chemicals (Sweden) to generate calibration curves for the methanolysis assay.

#### 2.5.2 Fourier-transform infrared spectroscopy

The infrared spectra were recorded in the range from 4,000 to 650 cm^−1^ with a Fourier Transform Spectrophotometer (FTIR) Nicolet iS10 (ThermoScientific, United States) by the attenuated total reflection method (ATR-FTIR) on samples without prior preparation. Each spectrum analyzed was the average of 16 recorded measurements.

#### 2.5.3 Differential scanning calorimetry and thermogravimetry

Differential scanning calorimetry (DSC) tests were carried out in a nitrogen atmosphere with a Q200 differential scanning calorimeter (TA Instruments, United States). The temperature ranged from −70 to 230°C with a heating/cooling rate of 10°C/min. Samples of approximately 1 mg were first rapidly heated to 230°C and conditioned for 1 min to remove the thermal history of the material. The samples were then cooled to −70°C and re-heated to 230°C. Thermal analysis of polymers was based on the second heating curve, where the glass transition temperature (T_g_), melting point (T_m_), and change in enthalpy of the melting process (ΔH_m_) were recorded.

Thermogravimetry (TG) tests were performed in a nitrogen atmosphere with a Q500 thermobalance (TA Instruments, United States). The temperature ranged from 25 to 700°C with a heating rate of 10°C/min. The mass of the analyzed samples was approximately 1 mg. From the thermogravimetric curve, 5% mass loss temperature (T_5%_) was determined and used as a parameter determining the onset of thermal degradation of the material, taken as the thermal resistance of the material (T_d_). The differential thermogravimetric curve (DTG) (first derivative of the TG curve) was also presented, and the T_max_ values determining the temperature of the fastest mass loss were also presented.

## 3 Results and discussion

### 3.1 Capability of *Pseudomonas* sp. strains to grow and produce PHAs in mineral salt media containing SMCFA_synthetic_-rich stream

A preliminary study of the *Pseudomonas* sp. GL01, *Pseudomonas* sp. GL06, and *P. antarctica* was conducted to determine their ability to grow and synthesize PHAs in the presence of a mixture of SMCFAs (SMCFA_synthetic_-rich stream) that mimicked the effluent from whey wastewater anaerobic fermentation as the carbon source.

**FIGURE 1:**
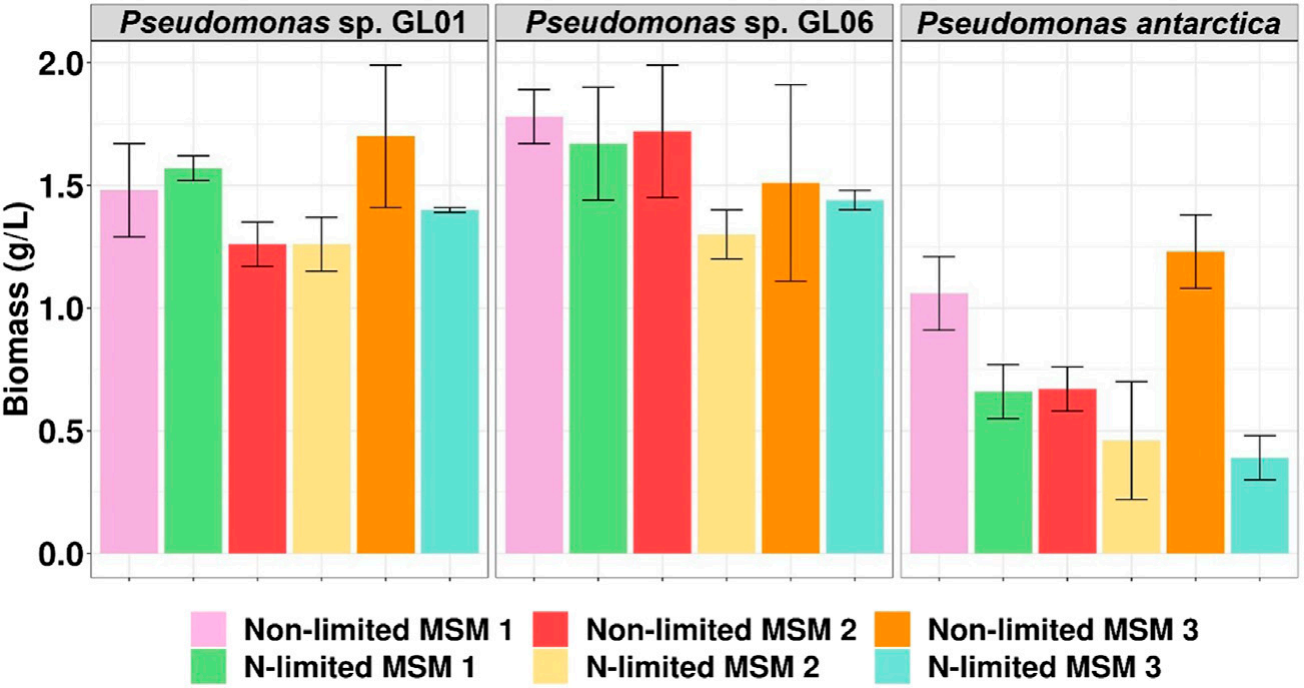
Biomass concentration (g/L) of *Pseudomonas* sp. Gl01, *Pseudomonas* sp. Gl06, and *P. antarctica* at 48 h cultured on three different growth media (MSM 1, MSM 2, and MSM 3) in shake flask experiments under non-limited and N-limited conditions using SMCFA_synthetic_-rich stream.

As can be seen in Figure 1 all tested bacteria could grow in the medium supplemented with SMCFA_synthetic_-rich stream under all culture conditions used. In particular, the *Pseudomonas* sp. GL06 showed the highest biomass concentration among all tested MSM media, achieving the maximum biomass value in MSM 1 (1.8 g/L of CDM). A slightly lower amount of CDM (1.7 g/L) was achieved in the cultivation of *Pseudomonas* sp. GL01 grown on MSM 2 under non-limited conditions. A 3-fold lower CDM of all *Pseudomonas putida* strains (KT2440, CA-3, and GO16) was determined by Cerrone et al. (2014) ,who cultured them using fatty acids derived from anaerobic digestion of grass. In our study, low growth was detected in the cultivation of *P. antarctica* in culture media under non-limited as well as nitrogen-limited conditions, that could suggest the inhibitory effect of the SMCFA_synthetic_-rich stream used. However, higher CDM values were observed in the cultures under non-limited conditions.

**FIGURE 2:**
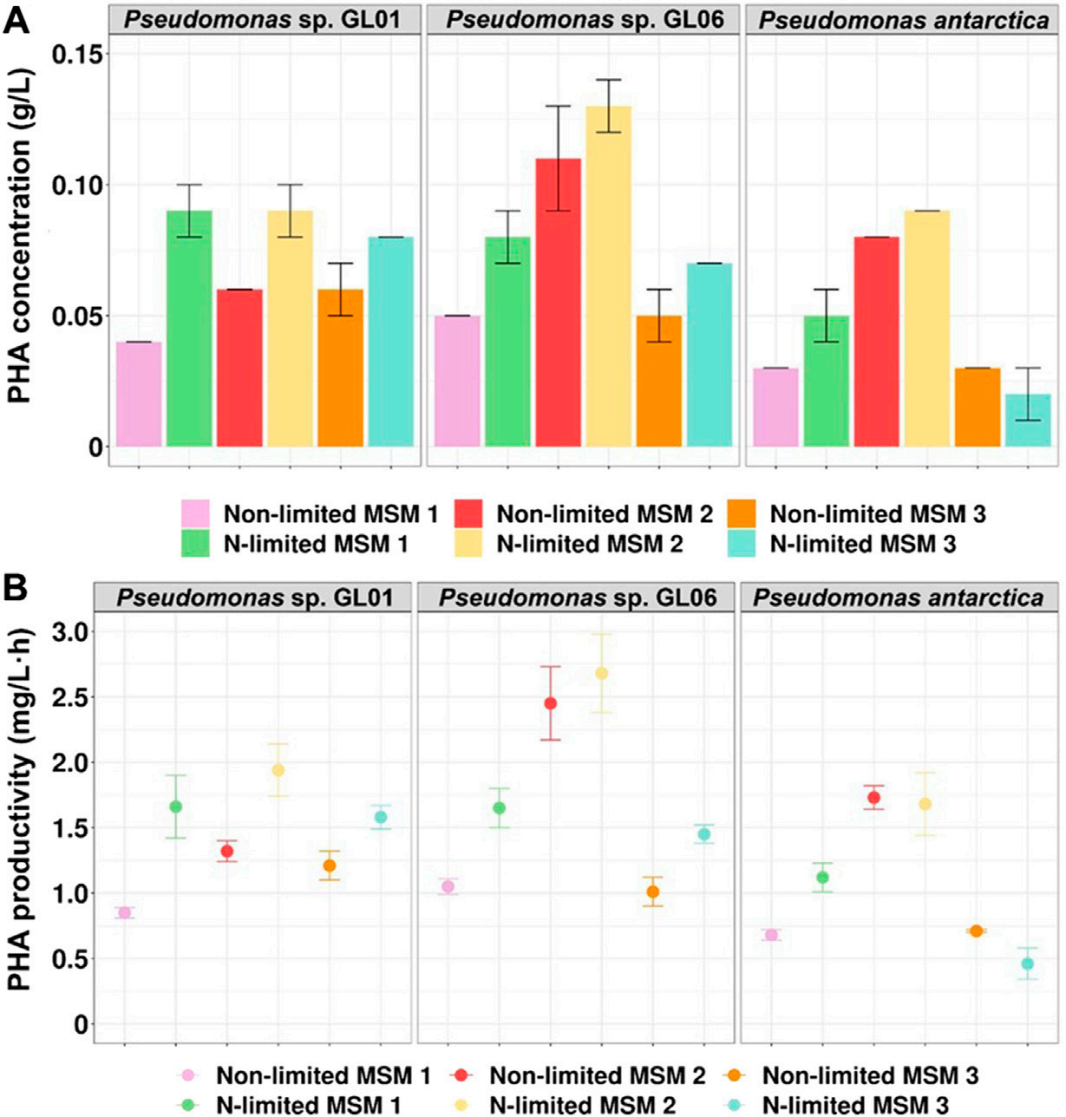
PHA production from SMCFA_synthetic_-rich stream by *Pseudomonas* sp. GL01, *Pseudomonas* sp. GL06, and *P. antarctica* at 48 h cultured on three different growth media (MSM 1, MSM 2, and MSM 3) in shake flask experiments under non-limited and N-limited conditions. **(A)** PHA concentration (g/L) and **(B)** PHA productivity (mg/L h).

In general, adequate nitrogen source is essential for bacterial cell growth. On the other hand, PHA synthesis and accumulation by bacteria belonging to *Pseudomonas* genera is induced by nitrogen limitation in the culture medium (Hoffmann and Rehm, 2005). Our results showed that PHA concentration and productivity increased under nitrogen starvation and depended on the type of MSM used. Only in the case of *P. antarctica* grown on MSM 3, PHA yield was not triggered by nitrogen limitation (Figures 2A,B). The best PHA concentration was achieved in the culture with *Pseudomonas* sp. GL06 grown on MSM 2 under N-limitation (0.13 g/L) at the PHA productivity level of 2.7 mg/L h. Aremu et al. (2021) reported a similar PHA value (0.12 g/L) in *Pseudomonas oleovorans* culture supplemented with acetic acid as the carbon source in a nitrogen-limited medium.

Furthermore, our results suggests that PHA production could be enhanced by controlling the nitrogen source. In all tested *Pseudomonas* strains, higher PHA efficiency was observed when MSM 2 [contained (NH_4_)_2_SO_4_] was used under both growth conditions compared to MSM 1 (consisted of NH_4_Cl). Moreover, our data revealed that when phosphate sources (Na_2_HPO_4_·7H_2_O and KH_2_PO_4_) were used in a higher concentration (MSM 2 compared to MSM 3), PHAs were synthesized and accumulated at higher yields, in particular by *Pseudomonas* sp. GL06 and *P. antarctica.* It could be concluded that the PHA concentration and productivity were dependent on the culture conditions used.

### 3.2 SMCFA_extracted_-rich stream derived from anaerobic fermentation as the carbon source for biomass growth and PHA production

**FIGURE 3:**
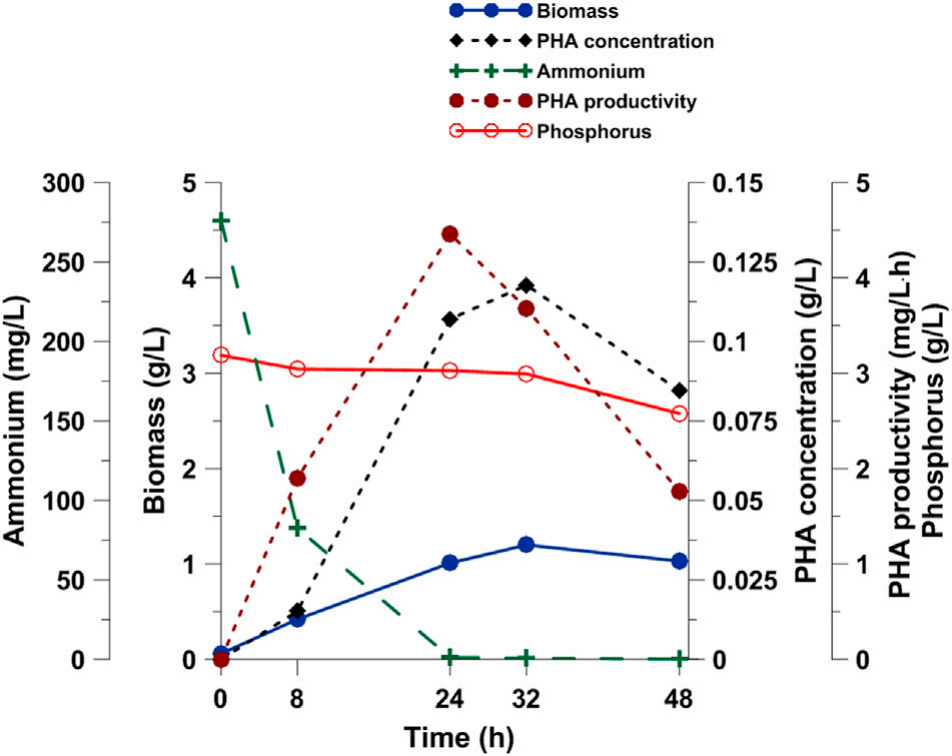
PHA production, cell, ammonium and phosphate concentration during *Pseudomonas* sp. GL06 cultivation on SMCFA_extracted_-rich stream in the bioreactor fermentation.

On the basis of the previously described results that *Pseudomonas* sp. GL06 produced PHAs with the highest efficiency, it was selected for further investigations into PHA production with SMCFA_extracted_-rich stream from the acidogenic anaerobic mixed culture fermentation of acid whey obtained from a cheese production line. The fermentation process in a bioreactor led us to precisely monitor the bioprocess due to larger volume for sampling. As may be observed from the data reported in Figure 3, biomass concentration had been increasing for up to 32 h, and then reached 1.03 g/L at the end of the bioprocess. Comparable biomass data (1.03 g/L) were reported in *P. oleovorans* culture supplemented with volatile fatty acid streams from chicken manure where acetate was the predominant acid (Aremu et al., 2021). Higher biomass concentration was determined by our research group by supplementation of the culture medium with the same SMCFA_extracted_-rich stream in the cultivation of *Paracoccus homiensis* (Szacherska et al., 2021). Moreover, our results confirmed that PHA productivity increased under nitrogen limitation, reaching a maximum value of 4.5 mg/L h at 24 h of the fermentation after ammonium exhaustion in the growth medium was reached. Similar results (4.4 mg/L h) were achieved by Kourmentza et al. (2009), who cultivated *Pseudomonas* sp. on a mixture of acetic, propionic, and butyric acids under nitrogen-limiting conditions. Goh and Tan (2012), by feeding of glucose and glycerol in the cultivation of arctic *Pseudomonas* sp. UMAB-40, determined a much lower productivity, i.e., 2.6 mg/L h and 1.6 mg/L h, respectively. Exponential nonanoic acid-limited growth of *P. putida* KT2440 resulted in a PHA content of 75.4%, giving a high cumulative PHA productivity of 1.11 g/L h (Sun et al., 2007). It should be emphasized that in our study, the PHA yield did not increase more after 24 h. The observed reduction in PHA synthesis can be explained by the consumption of the accumulated PHAs as energy reserves to extend the survival of bacteria after depletion of the carbon and nitrogen sources (Kourmentza et al., 2017). The same observation was made by Venkateswar Reddy et al. (2012) who reported that after reaching the maximum PHA concentration, the production capacity of *Pseudomonas otitidis* decreased up to the end of the experiment. The authors suggested that due to the famine phase, the stored PHAs were consumed by the bacteria to maintain their cell activity in the absence of essential nutrients. Furthermore, the PHA productivity was about 60% higher than that determined in the shake flask experiments using the same culture medium. It should be noted that the difference between SMCFA_synthetic_-rich stream and SMCFA_extracted_-rich stream was the presence of lactic acid and ethanol in the latter. Thus, our data suggested that neither lactic acid nor ethanol affected the PHA productivity negatively.

Furthermore, quite low PHA productivity was reported in the cultivation of *P. homiensis* grown on the same (as in our study) SMCFA_extracted_-rich stream, suggesting that the presence of butyric acid as the dominant component had more inhibitory effect due to the fact that a single short-chain-fatty acid in the medium resulted in the lowest PHA production efficiency compared with others (Szacherska et al., 2021).

### 3.3 The characterization of PHAs

#### 3.3.1 GC-MS

**FIGURE 4:**
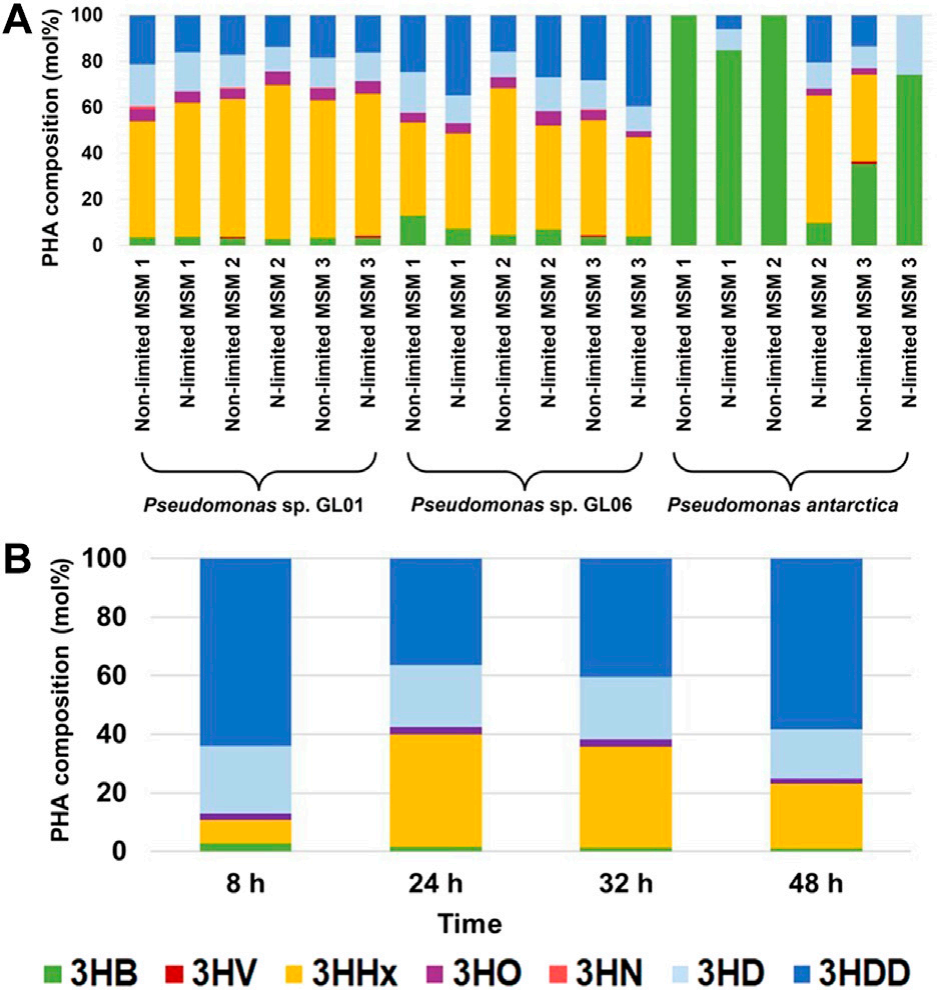
Monomeric composition of extracted PHAs from shake flasks **(A)** and bioreactor **(B)**.

As shown in Figure 4, the PHA monomeric composition varied with the bacterial strain and the type of the growth medium used. Our results proved that in the cultivations supplemented with SMCFA_synthetic-_rich stream that simulated a real SMCFA-rich stream, *Pseudomonas* sp. GL01 and GL06 showed the tendency to produce high amounts of 3-hydroxyhexanoate (3HHx) and 3-hydroxydodecanoate (3HDD) as the main monomers and lower content of 3-hydroxybutyrate (3HB), 3-hydroxyoctanoate (3HO), and 3-hydroxydecaonate (3HD). Interestingly, only in the cultivations of *Pseudomonas* sp. GL01 performed under non-limited growth conditions a trace amounts of 3-hydroxynonanoate was detected. This monomer was found mainly at the end of the fermentation of the same bacterial strain using saponified waste palm oil (Możejko and Ciesielski, 2013). The level of 3HB in the extracted PHAs seems to be strain dependent. Our results indicated that *P. antarctica* is able to synthesize P(3HB) homopolymer grown on MSM1 and MSM2 under non-limited conditions, whereas this bacterium cultured on MSM 3 under both conditions applied was capable of producing scl-mcl copolymers. *P. otitidis* also prefers to produce copolymers, however, consisting of 3HB and 3HV monomers (Venkateswar Reddy et al., 2012). The authors reported that the copolymer showed higher 3HB monomer content (91 mol%) when the bacteria were cultured on synthetic acids (acetate, propionate, and butyrate) compared to acidogenic effluent from the biohydrogen reactor. Furthermore, it was observed that *P. putida* KT2440 grown in the medium supplemented with acetate as the sole carbon source produced P(3HO-*co*-3HD-*co*-3HDD) terpolymer with 3HD as the principal monomer (Yang et al., 2019).

The GC/MS analysis confirmed that at the beginning of the cultivations of *Pseudomonas* sp. GL06 in the bioreactor, no PHAs were detected. The repeat-unit composition of the purified biopolymers extracted from the bioreactor at 8 h of the fermentation was found to consist mainly of 3HD and 3HDD and lesser amounts of 3HB, 3HHx, and 3HO. From 24 to 48 h of the cultivation higher content of 3HHx fraction was observed from 38.5 mol% to 22.1 mol%, respectively. 3HB and 3HO monomers were detected as minor components in extracted biomaterials. Similarly, when glucose, glycerol, and fructose were supplied as carbon sources to *Pseudomonas* sp. B14-6 by Choi et al. (2021), scl-mcl copolymer was detected. However, *P. putida* KT2440 and CA-3 synthesized mcl-copolymers cultured on the concentrated fatty acid mixture derived from the anaerobic digestion leachate. Cerrone et al. (2014) showed that these strains produced 3HD as the main constituent and 3HO and 3HDD as minor components.

#### 3.3.2 FTIR

**FIGURE 5:**
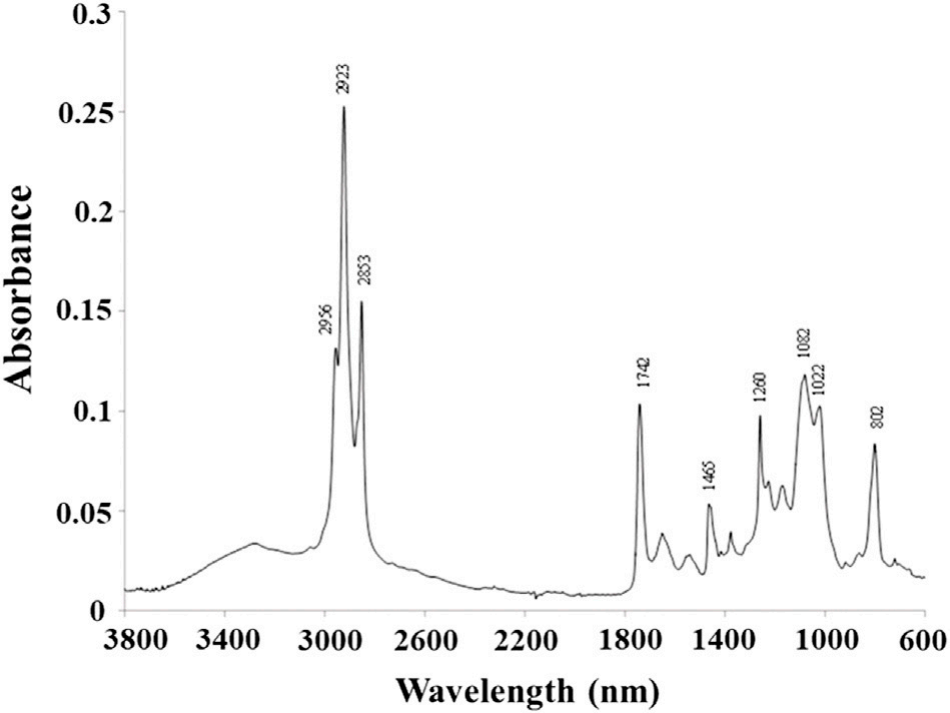
FTIR spectra of P(3HB-co-3HHx-co-3HO-co-3HD-co-3HDD) extracted from the *Pseudomonas* sp. GL06 cells at 48 h cultured in the bioreactor.

The FTIR spectra of the polymer produced is presented in Figure 5. The spectrum displays the typical bands for PHAs (Shamala et al., 2009; Rebocho et al., 2020; Meneses et al., 2021). Peaks at 2,956, 2,923, and 2,853 cm^−1^ can be assigned to the stretching vibration of C-H bonds of methyl (CH_3_) and methylene (CH_2_) groups of the PHA molecule. The high intensity of the 3,000–2,700 band (the highest in the entire spectrum) results from the presence of a large amount of medium chain length monomers in the tested polymer. The absorbance band located at 1740 cm^−1^ is attributed to the stretching vibration of the C=O group (ester carbonyl) in the PHA polyester. The bands between 1,022 and 1,260 cm^−1^ are related to the crystallinity of the material, with the 1,082 cm^−1^ peak attributed to C–O bonds. The band appearing at 802 cm^−1^ represents the C-C stretching bond and is also characteristic of PHA.

#### 3.3.3 Thermal analysis

**FIGURE 6:**
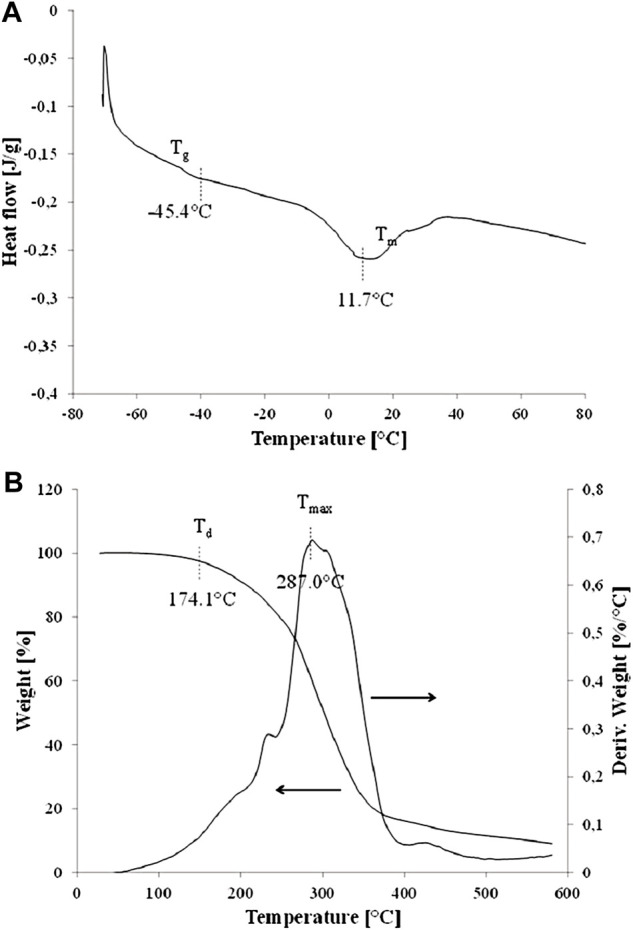
DSC curves **(A)** and TG curves **(B)** of P(3HB-co-3HHx-co-3HO-co-3HD-co-3HDD) extracted from the *Pseudomonas* sp. GL06 cells at 48 h cultured in the bioreactor.

As can be shown in Figure 6A, DSC studies proved that glass transition temperature (T_g_) of P(3HB-co-3HHx-co-3HO-co-3HD-co-3HDD) produced by *Pseudomonas* sp. GL01 reached −45.4°C. Furthermore, a small melting peak with a melting temperature (T_m_) of 11.7°C and enthalpy change (ΔH_m_) of 6.6 J/g was observed**.** The recorded T_g_ value is typical for mcl-PHAs and corresponds to the values reported in the literature (Simon-Colin et al., 2012; Basnett et al., 2017). The obtained melting point (11.7°C) is low and unusual, as in most cases the recorded T_m_ mcl-PHA values are in the range from 40 to 55°C. Lower T_m_ values are related to the low degree of crystallinity of this polymer and the predominance of the amorphous phase. The low content of the crystalline phase in the tested material is confirmed by the FTIR tests (low absorbance of peaks related to the crystalline phase) and the very low ΔH_m_ value obtained in the DSC tests. Nevertheless a P(3HO-*co*-3HD-*co*-3HDD-*co*-3HTD) copolymer produced by *Yarrowia lipolytica* ThYl_1,166 also possess low T_g_ (−39°C) and T_m_ (19°C) values indicating its amorphous behavior and flexibility at ambient temperature (Rigouin et al., 2019).

Moreover, the degradation temperature (T_d_) of the tested scl-mcl copolymer was observed at 174.1°C (Figure 6B) and was lower than the typical T_d_ values recorded for mcl-PHAs (above 200°C) (Simon-Colin et al., 2012; Basnett et al., 2017) due to its low degree of crystallinity. Additionally, TG analysis showed that the beginning of the degradation process already started just above 100°C and proceeded over a fairly large temperature range with the maximum intensity (T_max_) at 287.0°C (Figure 6B). The course of the degradation process may have been caused by a large number of macromolecules of shorter length (lower molecular weight), which are characterized by lower degradation temperatures. The fact that 3HB monomer was present in the extracted copolymer could be the reason for its lower thermal resistance.

## 4 Conclusion

Supplementation of synthetic SMCFAs in bacterial cultures would be expensive. Therefore using SMCFA-rich stream from the fermentation of agro-industrial waste to produce PHAs can significantly reduce the cost of the process. In this study, both variants of SMCFAs were proved to be potential substrates for PHA production by *Pseudomonas* strains. The highest PHA productivity was achieved in *Pseudomonas* sp. Gl06 cultivation in the bioreactor under N-limited with MSM 2 using SMCFA_extracted_-rich stream derived from MCF using food waste. The structure and thermal properties of the PHAs were analytically verified and we proved that the extracted scl-mcl copolymers possess lower melting point and degradation temperature compared to the PHAs described in the literature. These findings indicate the possibility of feeding the *Pseudomonas* sp. GL06 with cheap SMCFA_extracted_-rich stream to produce PHA copolymers with improved properties.

## Data Availability

The original contributions presented in the study are included in the article; further inquiries can be directed to the corresponding author.

## References

[B1] Akaraonye E. Keshavarz T. Roy I. (2010). Production of polyhydroxyalkanoates: The future green materials of choice. J. Chem. Technol. Biotechnol. 85, 732–743. 10.1002/jctb.2392

[B2] Albuquerque M. G. E. Eiroa M. Torres C. Nunes B. R. Reis M. A. M. (2007). Strategies for the development of a side stream process for polyhydroxyalkanoate (PHA) production from sugar cane molasses. J. Biotechnol. 130, 411–421. 10.1016/j.jbiotec.2007.05.011 17602776

[B3] Aremu M. O. Ishola M. M. Taherzadeh M. J. (2021). Polyhydroxyalkanoates (PHAs) production from volatile fatty acids (VFAs) from organic wastes by *Pseudomonas oleovorans* . Fermentation 7, 287. 10.3390/fermentation7040287

[B4] Basnett P. Lukasiewicz B. Marcello E. Gura H. K. Knowles J. C. Roy J. (2017). Production of a novel medium chain length poly(3-hydroxyalkanoate) using unprocessed biodiesel waste and its evaluation as a tissue engineering scaffold. Microb. Biotechnol. 10, 1384–1399. 10.1111/1751-7915.12782 28905518 PMC5658593

[B5] Beccari M. Bertin L. Dionisi D. Fava F. Lampis S. Majone M. (2009). Exploiting olive oil mill effluents as a renewable resource for production of biodegradable polymers through a combined anaerobic–aerobic process. J. Chem. Technol. Biotechnol. 84, 901–908. 10.1002/jctb.2173

[B6] Bengtsson S. Werker A. Christensson M. Welander T. (2008). Production of polyhydroxyalkanoates by activated sludge treating a paper mill wastewater. Bioresour. Technol. 99, 509–516. 10.1016/j.biortech.2007.01.020 17360180

[B7] Cerrone F. Choudhari S. K. Davis R. Cysneiros D. O’Flaherty V. Duane G. (2014). Medium chain length polyhydroxyalkanoate (mcl-PHA) production from volatile fatty acids derived from the anaerobic digestion of grass. Appl. Microbiol. Biotechnol. 98, 611–620. 10.1007/s00253-013-5323-x 24162086

[B8] Choi T. R. Park Y. L. Song H. S. Lee S. M. Park S. L. Lee H. S. (2021). Fructose-based production of short-chain-length and medium-chain-length polyhydroxyalkanoate copolymer by arctic *Pseudomonas* sp. B14-6. Polymers 13, 1398. 10.3390/polym13091398 33925903 PMC8123457

[B9] Ciesielski S. Możejko J. Przybyłek G. (2010). The influence of nitrogen limitation on mcl-PHA synthesis by two newly isolated strains of *Pseudomonas* sp. J. Ind. Microbiol. Biotechnol. 37, 511–520. 10.1007/s10295-010-0698-5 20204456

[B10] Duber A. Jaroszynski Ł. Zagrodnik R. Chwialkowska J. Juzwa W. Ciesielski S. (2018). Exploiting the real wastewater potential for resource recovery – N-Caproate production from acid whey. Green Chem. 20, 3790–3803. 10.1039/c8gc01759j

[B11] Erickson B. E. (2017). Acid whey: Is the waste product an untapped goldmine? Chem. Eng. News. 95, 26–30. 10.1021/cen-09506-cover

[B12] Goh Y. S. Tan I. K. P. (2012). Polyhydroxyalkanoate production by Antarctic soil bacteria isolated from casey station and signy island. Microbiol. Res. 167, 211–219. 10.1016/j.micres.2011.08.002 21945102

[B43] Hoffmann N. Rehm B. H. (2005). Nitrogen-dependent regulation of medium-chain length polyhydroxyalkanoate biosynthesis genes in pseudomonads. Biotechnol. Lett. 27, 279–282. 10.1007/s10529-004-8353-8 15742151

[B13] Koller M. Braunegg G. (2015). Biomediated production of structurally diverse poly(hydroxyalkanoates) from surplus streams of the animal processing industry. Polimery 60 (5), 298–308. 10.14314/polimery.2015.298

[B14] Kourmentza C. Ntaikou I. Kornaros M. Lyberatos G. (2009). Production of PHAs from mixed and pure cultures of *Pseudomonas* sp. using short-chain fatty acids as carbon source under nitrogen limitation. Desalination 248, 723–732. 10.1016/j.desal.2009.01.010

[B15] Kourmentza C. Plácido J. Venetsaneas N. Burniol-Figols A. Varrone C. Gavala H. N. (2017). Recent advances and challenges towards sustainable polyhydroxyalkanoate (PHA) production. Bioeng. (Basel). 4 (2), 55. 10.3390/bioengineering4020055 PMC5590474 28952534

[B16] Lad B. C. Coleman S. M. Alper H. S. (2022). Microbial valorization of underutilized and nonconventional waste streams. J. Ind. Microbiol. Biotechnol. 49, kuab056. 10.1093/jimb/kuab056 34529075 PMC9118980

[B17] Liu C. H. Chen H. Y. LeeChen Y. L. Sheu D. S. (2021). The polyhydroxyalkanoate (PHA) synthase 1 of *Pseudomonas* sp. H9 synthesized a 3-hydroxybutyrate-dominant hybrid of short- and medium-chain-length PHA. Enzyme Microb. Technol. 143, 109719. 10.1016/j.enzmictec.2020.109719 33375979

[B18] Martinez G. A. Bertin L. Scoma A. Rebecchi S. Braunegg G. Fava F. (2015). Production of polyhydroxyalkanoates from dephenolised and fermented olive mill wastewaters by employing a pure culture of *Cupriavidus necator* . Biochem. Eng. J. 97, 92–100. 10.1016/j.bej.2015.02.015

[B19] Meneses L. Craveiro R. Jesus A. R. Reis M. A. M. Freitas F. Paiva A. (2021). Supercritical CO_2_ assisted impregnation of ibuprofen on medium-chain-length polyhydroxyalkanoates (mcl-PHA). Molecules 26, 4772. 10.3390/MOLECULES26164772 34443357 PMC8400196

[B20] Możejko J. Ciesielski S. (2013). Saponified waste palm oil as an attractive renewable resource for mcl-polyhydroxyalkanoate synthesis. J. Biosci. Bioeng. 116, 485–492. 10.1016/j.jbiosc.2013.04.014 23706994

[B42] Możejko-Ciesielska J. Pokój T. (2018). Exploring nutrient limitation for polyhydroxyalkanoates synthesis by newly isolated strains of *Aeromonas* sp. using biodiesel-derived glycerol as a substrate. PeerJ 6, e5838. 10.7717/peerj.5838 30370188 PMC6202957

[B21] Mozejko-Ciesielska J. Szacherska K. Marciniak P. (2019). *Pseudomonas* species as producers of eco-friendly polyhydroxyalkanoates. J. Polym. Environ. 27, 1151–1166. 10.1007/s10924-019-01422-1

[B22] Munir S. Jamil N. (2018). Polyhydroxyalkanoates (PHA) production in bacterial co-culture using glucose and volatile fatty acids as carbon source. J. Basic Microbiol. 58 (3), 247–254. 10.1002/jobm.201700276 29314110

[B23] Patel S. K. S. Singh M. Kumar P. Purohit H. J. Kalia V. C. (2012). Exploitation of defined bacterial cultures for production of hydrogen and polyhydroxybutyrate from pea shells. Biomass Bioenergy 36, 218–225. 10.1016/j.biombioe.2011.10.027

[B24] Poblete-Castro I. Escapa I. Jager C. Puchalka J. Lam M. C. Schomburg D. (2012). The metabolic response of *P. putida* KT2442 producing high levels of polyhydroxyalkanoate under single- and multiple-nutrient-limited growth: Highlights from a multi-level omics approach. Microb. Cell. Fact. 11, 34. 10.1186/1475-2859-11-34 22433058 PMC3325844

[B25] Rebocho A. T. Pereira J. R. Neves L. A. Alves V. D. Sevrin C. Grandfils C. (2020). Preparation and characterization of films based on a natural P(3HB)/mcl-PHA blend obtained through the Co-culture of *cupriavidus necator* and *Pseudomonas citronellolis* in apple pulp waste. Bioeng. (Basel). 7, 34. 10.3390/BIOENGINEERING7020034 PMC7356164 32260526

[B26] Reddy S. V. Thirumala M. Mahmood S. K. (2008). Production of PHB and P (3HB-co-3HV) biopolymers by *Bacillus megaterium* strain OU303A isolated from municipal sewage sludge. World J. Microbiol. Biotechnol. 25, 391–397. 10.1007/s11274-008-9903-3

[B27] Rigouin C. Lajus S. Ocando C. Borsenberger V. Nicaud J. M. Marty A. (2019). Production and characterization of two medium-chain-length polydroxyalkanoates by engineered strains of *Yarrowia lipolytica* . Microb. Cell. Fact. 18, 99. 10.1186/s12934-019-1140-y 31151440 PMC6545009

[B28] Shamala T. R. Divyashree M. S. Davis R. Kumari K. S. L. Vijayendra S. V. N. Raj B. (2009). Production and characterization of bacterial polyhydroxyalkanoate copolymers and evaluation of their blends by Fourier transform infrared spectroscopy and scanning electron microscopy. Indian J. Microbiol. 49, 251–258. 10.1007/S12088-009-0031-Z 23100778 PMC3450024

[B29] Simon-Colin C. Gouin C. Lemechko P. Schmitt S. Senant A. Kervarec N. (2012). Biosynthesis and characterization of polyhydroxyalkanoates by *Pseudomonas guezennei* from alkanoates and glucose. Int. J. Biol. Macromol. 51, 1063–1069. 10.1016/J.IJBIOMAC.2012.08.018 22947450

[B30] Solaiman D. K. Ashby R. D. (2005). Genetic characterization of the poly(hydroxyalkanoate) synthases of various *Pseudomonas oleovorans* strains. Curr. Microbiol. 50, 329–333. 10.1007/s00284-005-4508-7 15968501

[B31] Sun Z. Ramsay J. A. Guay M. Ramsay B. A. (2007). Carbon-limited fed-batch production of medium-chain-length polyhydroxyalkanoates from nonanoic acid by *Pseudomonas putida* KT2440. Appl. Microbiol. Biotechnol. 74, 69–77. 10.1007/s00253-006-0655-4 17063330

[B32] Szacherska K. Moraczewski K. Rytlewski P. Czaplicki S. Ciesielski S. Oleskowicz-Popiel P. (2021). Polyhydroxyalkanoates production from short and medium chain carboxylic acids by *Paracoccus homiensis* . Sci. Rep. 12 (1), 7263. 10.1038/s41598-022-11114-x PMC9068790 35508573

[B33] Tan D. Wang Y. Tong Y. Chen G. Q. (2021). Grand challenges for industrializing polyhydroxyalkanoates (PHAs). Trends Biotechnol. 39 (9), 953–963. 10.1016/j.tibtech.2020.11.010 33431229

[B34] Tan G. Y. A. Chen C. L. Li L. Ge L. Wang L. Razaad I. M. N. (2014). Start a research on biopolymer polyhydroxyalkanoate (PHA): A review. Polymers 6 (3), 706–754. 10.3390/polym6030706

[B35] Thomas C. M. Kumar D. Scheel R. Ramarao B. Nomura C. T. (2022). Production of Medium Chain Length polyhydroxyalkanoate copolymers from agro-industrial waste streams. Biocatal. Agric. Biotechnol. 43, 102385. 10.1016/j.bcab.2022.102385

[B36] Venkateswar Reddy M. Mawatari Y. Onodera R. Nakamura Y. Yuka Yajima Y. Chang Y. C. (2017). Polyhydroxyalkanoates (PHA) production from synthetic waste using *Pseudomonas pseudoflava*: PHA synthase enzyme activity analysis from. P. pseudoflava P. palleronii. Bioresour. Technol. 234, 99–105. 10.1016/j.biortech.2017.03.008 28319778

[B37] Venkateswar Reddy M. Nikhil G. N. Venkata Mohan S. Swamy Y. V. Sarma P. N. (2012). *Pseudomonas otitidis* as a potential biocatalyst for polyhydroxyalkanoates (PHA) synthesis using synthetic wastewater and acidogenic effluents. Bioresour. Technol. 123, 471–479. 10.1016/j.biortech.2012.07.077 22940357

[B38] Venkateswar Reddy M. Venkata Mohan S. (2012). Influence of aerobic and anoxic microenvironments on polyhydroxyalkanoates (PHA) production from food waste and acidogenic effluents using aerobic consortia. Bioresour. Technol. 103, 313–321. 10.1016/j.biortech.2011.09.040 22055090

[B39] Vu D. H. Wainaina S. Taherzadeh M. J. Åkesson D. Ferreira J. A. (2021). Production of polyhydroxyalkanoates (PHAs) by *Bacillus megaterium* using food waste acidogenic fermentation-derived volatile fatty acids. Bioengineered 12 (1), 2480–2498. 10.1080/21655979.2021.1935524 34115556 PMC8806590

[B40] Yang S. Li S. Jia X. (2019). Production of medium chain length polyhydroxyalkanoate from acetate by engineered *Pseudomonas putida* KT2440. J. Ind. Microbiol. Biotechnol. 46 (6), 793–800. 10.1007/s10295-019-02159-5 30864026

[B41] Zubairi S. I. Mantalaris A. Bismarck A. Aizad S. (2016). Polyhydroxyalkanoates (PHAs) for tissue engineering applications: Biotransformation of palm oil mill effluent (pome) to value-added polymers. J. Teknol. 78, 13–29. 10.11113/jt.v78.4042

